# Physiotherapists’ challenges with implementing the policy to Screen, Identify, Assess, and Support learners with physical disabilities

**DOI:** 10.4102/ajod.v15i0.1881

**Published:** 2026-03-03

**Authors:** Makwena M. Sibuyi, Desmond Mathye, Muziwakhe D. Tshabalala, Nombeko Mshunqane

**Affiliations:** 1 Department of Physiotherapy, Faculty of Health Science, Sefako Makgatho Health Science, Tshwane, South Africa; 2 Department of Physiotherapy, Faculty of Health Sciences, University of Pretoria, Tshwane, South Africa

**Keywords:** SIAS policy, physical disabilities, physiotherapists, inclusive education, Rationalisation and Redeployment policy, School-Based Support Team, District-Based Support Team

## Abstract

**Background:**

Challenges persist in implementing the policy to Screen, Identify, Assess, and Support (SIAS) individuals with physical disabilities, particularly in rural provinces of South Africa. The challenges are compounded by the existing imbalance in the distribution of physiotherapists where the majority work in the health sector. However, physiotherapists are well-equipped to assess functional limitations and adapt school environments to support inclusive learning and participation.

**Objectives:**

The study explored the challenges physiotherapists experienced in implementing the policy to SIAS learners with physical disabilities.

**Method:**

Seven physiotherapists employed by the provincial Department of Education participated in a qualitative, single exploratory case study utilising virtual focus group discussions. Data were analysed with a six-step approach to inductive thematic data analysis on ATLAS.ti version 19 software.

**Results:**

Two overarching themes with sub-themes emerged: (1) Poor knowledge of the SIAS policy as a result of lack of in-service training and fear of transfers through the Rationalisation and Redeployment policy; and (2) lack of interprofessional collaboration because of unclear roles and responsibilities, and absence of support structures with regard to the School-Based Support Teams, Circuit-Based Support Teams, and District-Based Support Teams.

**Conclusion:**

Ongoing in-service training, defining roles and responsibilities of physiotherapists and improving the functioning of support structures are essential for effective policy implementation.

**Contribution:**

The study bridges the gap in research on the participation of physiotherapists within the SIAS policy framework. Participation of physiotherapists would optimise support for learners and improve educational outcomes.

## Introduction

Education White Paper 6 (EWP6), titled ‘Special Needs Education-Building an Inclusive Education and Training System’, was established to address past discriminatory practices, which resulted in less than 20% of children with disabilities accessing education (Department of Basic Education [DBE] 2001, 2015). The constitution for the Republic of South Africa 1996 recognises access to education as a basic human right for all. Therefore, the endorsement of the EWP6 by the Department of Basic Education seek to establish equity, access to quality education, and support for learners, parents, and communities in overcoming barriers to learning (DBE 2001). According to the National Development Plan 2030, access to quality education for children with disabilities will mobilise South Africa to achieve its employment equity goals (National Planning Commission 2012).

Prior to 2014, in South Africa, teachers used their intuition and the deficit model to identify learners with learning barriers (Mkhuma, Maseko & Tlale 2014). Learners were admitted to schools based on their successful aptitude tests results. This system stigmatised learners who did not pass aptitude tests as it failed to consider the innate learning style of the learner (Mkhuma et al. 2014). To facilitate educational reforms, the policy to Screen, Identify, Assess, and Support (SIAS) learners with learning barriers was implemented in 2014 after it was piloted at various schools in 2008 (Engelbrecht, Oswald & Forlin 2006). The policy dispenses guidelines on how learners with barriers to learning can be assessed and supported to prevent high dropout rates (DBE 2014).

The SIAS process of assessment ensures consistent procedures in assessing and providing support by utilising toolkits such as Learner Profiles, Special Needs Assessment 1 (SNA1), Special Needs Assessment 2 (SNA2), Special Needs Assessment 3 (SNA3), and Individual Support Plans (ISPs) (DBE 2014). These assessments focus on identifying barriers to learning, level of function, and participation rather than academic achievement. The class educator uses the Learner Profile to screen and identify learners that have barriers across social, physical or academic domains. Supplementary material from *Road to Health* booklet and reports from therapists including physiotherapists and parents assist the educator in identifying learners at risk of not benefitting from academic activities. The educator becomes a case manager and utilises the SNA1 to facilitate support by collaborating with the parents and the learner (above 12 years). The educator should approach the School-Based Support Team (SBST) when the planned support is not benefitting the learner. The SBST utilising the SNA2 reviews the support provided by the educator and makes recommendations to support the educator and the learner. Where available, therapists based at schools including physiotherapists form part of the SBST to develop ISPs for the identified at-risk learners. It is crucial for the ISP to be monitored and evaluated for progress by the class educator and physiotherapists, to make necessary adjustments to the intervention or to make a referral to the District-Based Support Team (DBST). This team comprises of inclusive education specialists and therapists based at the district level.

Therefore, the role of the physiotherapist is implied at the school level and the district level. This process with the involvement of physiotherapists may lead to the referral of some learners to either full-service or mainstream schools, ultimately contributing to a decrease in the waiting list for learners currently unable to access special education. It is noteworthy that the SIAS policy is closely linked with the Rationalisation and Redeployment (R&R) policy. The primary objective of the R&R policy is to establish equity in the allocation of educator posts (DBE 1995). This policy stipulates that educators who are in excess at one school should be redeployed to other schools that experience a shortage based on the learner-to-educator ratio. This ratio is 40:1 for primary and 35:1 for high schools (Nemutandani 2004). As more learners are transferred to other schools without the admission of new learners, it becomes increasingly likely that some educators will need to be redeployed.

As indicated in the policy document, therapists’ contribution often depends on their availability at schools (DBE 2014). This is a challenging arrangement that requires policy review because physiotherapists working in schools promote inclusive education through the transfer of their knowledge and expertise, conduct functional assessments of activity limitations and participation restrictions within the context of the learner. Essentially, physiotherapists in education play a crucial role in the early detection and redressal of learning barriers attributable to motor skills (Hatch & Dombrowski 2019). Gross motor skills have been found to significantly bolster visual-motor integration, which contributes positively to a child’s readiness for school (Oberer, Gashaj & Roebers 2018). Through interprofessional collaboration with other stakeholders at the school, physiotherapists contribute to setting rehabilitation and education goals to develop support plans that are customised for every learner (Pillay & Tlale 2024).

Although the SIAS policy document does not explicitly emphasise the role of school physiotherapists in the assessment process, their inadequate understanding of the challenges they face in implementing the SIAS policy to support learners with physical disabilities has not been reported according to the first author’s knowledge. Understanding these challenges is essential in improving support structures and ensuring effective inclusion of physiotherapists in the school setting.

## Research methods and design

In this qualitative descriptive study, we employed an interpretivist theory otherwise known as interpretivism. Interpretivism assumes that reality is subjective, multiple and socially constructed (Kumatongo & Muzata 2021). We can only understand someone’s reality through their experience of that reality, which may be different from another person’s shaped by the individuals’ historical or social perspective. The use of interpretivism allows multiple exploration of the various dimensions of the truth.

### Study design

This is a qualitative, single exploratory case study design using focus group discussions. In addition to the benefits of qualitative exploration of reality, the small number of education physiotherapists in the South African special needs schools makes focus group discussion a suitable approach to exploring subject of this study.

### Population and sampling

The study was conducted in the Limpopo province, which is one of South Africa’s rural provinces. Limpopo province is the fifth largest province out of nine provinces in South Africa in terms of population size (South African Institute of Race Relations 2020). The population is predominantly black African (96.7%), and highly varied in terms of languages spoken. The majority speaks Sepedi (52.9%), followed by Xitsonga (17%) and Tshivenda (16.7%) (Limpopo Department of Education 2020). As a result of the province being rural, there are constraints pertaining to resource allocation. For instance, the number of public special schools and healthcare facilities differs significantly from those in Gauteng province, which is urban. Limpopo province only has 35 special schools accommodating learners with intellectual and physical disabilities including visual and hearing impairments whereas there are 120 special schools in Gauteng province (Gauteng Department of Education 2023). Of the 35 special schools, only three are designated for learners with physical disabilities and with only one admitting learners from Grade R to Grade 12.

During the study period, the Limpopo Department of Education employed eight education physiotherapists, including researchers. Consequently, a sample of seven education physiotherapists (*n* = 7) working for the Limpopo Department of Education were purposefully sampled (Palinkas et al. 2015) to participate in the study. The purposive sampling approach enabled the first author to select participants who had experience and knowledge related to the research question and the objective of the study. Participants who are knowledgeable could provide in-depth information to deepen the understanding pertaining to challenges experienced with supporting learners with physical disabilities. Of the seven, only one physiotherapist was based at a special school, while six were appointed on an itinerant basis, rendering services to various education districts.

### Inclusion criteria

Physiotherapists working in the school setting were eligible to participate in the study, irrespective of their years of experience.

### Recruitment

Following ethical approval from the provincial Department of Education, prospective participants were emailed detailed information letters and informed consent forms individually to invite them to participate in the focus group discussion for the study. The recruitment process started on the 19th of April 2021 and continued till the 06th of May 2021. Subsequently, after reminders all education physiotherapists expressed their interest and provided written informed consent forms via emails. Table 1 shows the demographic characteristics of the seven participants. The median age and years of experience were > 40 years and 11–15 years, respectively.

**TABLE 1:** Demographic characteristics of participants (*N* = 7).

Gender	Code	Age range (years)	Highest qualification	Occupation	Experience (years)
Female	P1	> 40	PhD	Physiotherapist – District-based	11–15
Female	P2	25–30	BSc (Physiotherapy)	Physiotherapist – School-based	< 5
Male	P3	31–40	BSc (Physiotherapy)	Physiotherapist – District-based	5–10
Male	P4	> 40	MSc (Physiotherapy)	Physiotherapist – District-based	11–15
Male	P5	> 40	BSc (Physiotherapy)	Physiotherapist – District-based	11–15
Female	P6	31–40	BSc (Physiotherapy)	Physiotherapist – District-based	5–10
Female	P7	> 40	BSc (Physiotherapy)	Physiotherapist – District-based	11–15

PhD, Doctor of Philosophy; BSc, Bachelor of Science; MSc, Master of Science.

### Data collection

Considering the geographical dispersion of participants, a virtual session was adopted to facilitate convenient engagement (Keen, Lomeli-Rodriguez & Joffe 2022). Data collection followed a respondent-moderated synchronous virtual format for focus group discussion conducted via Microsoft Teams® platform (Keemink et al. 2022). This approach involved participants choosing a moderator to facilitate the discussions using the interview guide while the first author (Makwena M. Sibuyi) was the moderator’s assistant during the discussion. In addition, the moderator ensured maximum participation from all participants.

This approach is thought to increase wide-ranging responses that are truthful from participants (Kamberelis & Dimitriadis 2005; Onwuegbuzie & Frels 2015). The moderator was taken through the literature that provided guidance and tips on how to conduct focus group discussions. These included being able to listen to participants, probe during discussions, and contain the discussions (Fusch, Fusch & Ness 2022). The moderator was also an employee in the provincial Department of Education and tasked with advancing inclusive education policies such as the SIAS policy. The provincial Department of Education in Limpopo provided a formal in-service training to education physiotherapists in which the moderator and other participants were involved. Although not all education physiotherapists received this formal training, they were involved in executing their responsibilities to conduct in-service training to educators in special schools about the SIAS policy.

In addition to the formal training and training the educators, the first author (Makwena M. Sibuyi) provided a mock interview session with the moderator (chosen by participants) and offered feedback on areas for improvement, such as making use of prompts to break the ice or starting the conversation, probing to allow the participant to elaborate on statements uttered, maintaining focus of the interview guide, constructive engagement instead of veering off the topic and managing time effectively.

The interview guide (Jamshed 2024) was shared with participants to familiarise themselves with the questions in preparation for responses and for participants to make changes with the questions where necessary. There was no indication from the participants that questions needed to be added or changed. Participants were asked about challenges experienced in implementing the SIAS policy. To ensure confidentiality, each participant opted for a pseudonym to safeguard their identity (Kaiser 2009). Participants chose these names: *Montsho, Celine, Sbakuza, Funky, R.S., Valentine, and Milkshake*. Before a Focus Group Discussion (FGD), all participants agreed upon the rules governing FGD. One FGD was conducted in English, audio recorded and lasted for 90 min.

### Data analysis

Inductive thematic analysis on ATLAS.ti version 9 was used to explore challenges experienced by education physiotherapists with the SIAS policy. Audio records were transcribed verbatim. Data were coded following a six-stage approach to inductive thematic analysis process (Dawadi 2021) by authors Makwena M. Sibuyi and Muziwakhe D. Tshabalala. Data saturation was reached when new data became redundant and participants did not have differing views (Francis et al. 2010). The six-steps began with the authors familiarising themselves with the data through repeated readings and note-taking. Initial codes were generated from quotations, which were then grouped into categories and refined into themes following consensus. The criteria for selecting quotations to be used for reporting were based on inclusive and ethical considerations involving a mix of quotations from different participants per theme where possible. Selected quotes that represented participants’ intentions were chosen to ensure that their meanings were not distorted. Personal identifiable information was removed to safeguard participant confidentiality (Brennan 2022).

Using a co-coder was aimed at maintaining coding consistency and validating the accuracy of the coding process. A consensus agreement was used as a strategy to determine themes (Prieto-González et al. 2021). This strategy allowed back-and-forth and in-depth discussions of themes among the authors yielding credible results. Finally, the two themes formed the basis of the study’s results and discussion.

### Trustworthiness

Strategies such as credibility, reflexivity, dependability, confirmability, and transferability ensured study rigour (Johnson, Adkins & Chauvin 2020).

Credibility was upheld through member checks where participants received transcripts to validate their accuracy (McKim 2023). In demonstrating reflexivity, the first author (Makwena M. Sibuyi) has a good understanding of the education sector, having worked for 4 years within the Inclusive Education sub-directorate of the provincial Department of Education and thus able to interpret participants’ responses accurately within the context. Prior collaboration with other physiotherapists and having trained educators in the province on SIAS policy makes the first author acknowledge her potential influence on the interpretation of data.

In interpreting the study’s findings, the first author considered the possible variability in participants’ experiences; only one directly working within the special school. It is possible that participants may have possessed a sense of compulsion to participate in this study, despite being informed of their right to withdraw from the study without consequences. Nonetheless, participants were contacted through their personal emails to receive information letters. It was hoped that communication through their personal emails would remove the sense of compulsion to participate in the study. However, in the first author’s reflection on the attitude and composures of the participants, throughout the study period, there was nothing to suggest that the participants were under any form of compulsion.

At the end of the focus group discussion, Makwena M. Sibuyi and the moderator allowed participants to reflect on questions and responses provided. Participants realised that they (School-based physiotherapist and those at district-based) had similar challenges in the education sector. It was then resolved that ongoing meetings were needed to find solutions collaboratively to the identified challenges. These meetings would take place for the first time since the appointment of educational physiotherapists at the district level. This showed a discussion like this was needed pointing to this study having relevance.

The researcher’s (Makwena M. Sibuyi) role entailed collaborative efforts with physiotherapists across various education districts, collectively working towards enhancing inclusive education. The first author was integral in educating educators from both special and full-service schools and training caregivers and Special Care Centres’ managers on inclusive education policies, with a particular focus on SIAS policy.

To ensure transferability, descriptions of the province name, the district names, participants’ demographics, data collection procedures, and duration were provided (Johnson et al. 2020). The findings are dependable, should the same interview guide, participants and similar context be used for another study. Lastly, confirmability was upheld through collaborative efforts with a co-coder, ensuring consensus and resolving any coding discrepancies that arose during the process (Nowell et al. 2017).

### Ethical considerations

Approval to conduct the study was granted by both the University Health Sciences Ethics Committee (Ref: 668/2020) and the provincial Department of Education to ensure ethical considerations were met. The research adhered to the ethical principles of conducting studies involving human participants, following the guidelines in the Helsinki Declaration 2013 version (General Assembly of the World Medical Association 2014). All participants signed informed consent forms, thus agreeing to participate in the study and to have their voices recorded for the interviews.

## Results

The study presents challenges experienced in implementing the policy to SIAS learners with physical disabilities. Table 2 presents themes, sub-themes, and participants’ quotations.

**TABLE 2:** Identified themes related to the challenges with Screen, Identify, Assess, and Support policy implementation.

Theme	Sub-theme	Quotations
Poor knowledge of the SIAS policy	-	‘I am saying even the initial procedure of learners having Learner Profiles is not executed, we struggled to have Learner Profiles … there were no Learner Profiles in place. I came in thinking I will be intervening at the Special Need Assessment 2, where I will look at the Special Need Assessment 2 forms for those learners needing support … but I found that I am talking Greek!’ (P1)
	A lack of in-service training	‘The biggest thing I have observed is.how familiar are we as therapists or teachers dealing with the kids directly in the school? I think it is clear now that I was not trained … because we were not part of the bigger group when we joined the team. I think maybe that is the reason why … those of us who joined the team later did not receive such training.’ (P5) ‘We did not receive any training regarding the SIAS … I self-acquainted myself with it.’ (P6) ‘Yes, I received a bit of a training.’ (P3) ‘I don’t remember if it was training or I just read through …’ (P7)
	Fear of transfers through the Rationalisation and Redeployment (R&R) Policy	‘There is going to be a low intake at the schools … then someone up there [*management*] … told the teacher it is like if … you go and continue with this process, it means all the kids here are going out of the system and when R&R comes, they are going [*teachers*] to be the first ones to be removed from the school … so they won’t implement that because the moment they implement, some will be removed … so they don’t want that. [*This*] is what I have gathered where I work.’ (P2)
A lack of interprofessional collaboration	A lack of role and responsibilities	‘You will never even see me anywhere. It means some of the decisions are taken maybe without my consult and some of those are my areas of specialty … I think that is the challenge.’ (P2) ‘At the beginning, you are not involved. You will be involved at the end of the process. [*Moreover*], you might even realise that if I had been involved at the beginning, that case might not even be there. For instance, the child might not be in that particular school.’ (P3) ‘Now, for as long as at a school level…or let me say the School-Based Support Team, they don’t understand at what point should we call a physio or any other health professional, that problem will remain. I think that is the main problem.’ (P3) ‘Especially our role as District-Based Support Team is not clear … do you understand? [*We*] were confused … because if you get to a school, they will be expecting you to do everything.’ (P4)
	A lack of support structures	‘When a school needs to go from the school level to the district level, they don’t even consult the circuit. You can see there’s a problem with how it’s being put into action.’ (P3) ‘Another issue was that in many cases, there’s no District-Based Support Team. So, even if teachers try to use the SIAS toolkits, nothing really happens.’ (P7)

SIAS, Screen, Identify, Assess, and Support.

### Theme 1: Poor knowledge of the Screen, Identify, Assess, and Support policy

There was a strong consensus among all the participants that poor knowledge of the SIAS policy on the part of both the educators and physiotherapists was a major challenge to the implementation of the SIAS policy. There were two factors that contributed to the poor knowledge of the SIAS policy. They included lack of in-service training and fear of transfers through the R&R policy.

### Theme 2: A lack of interprofessional collaboration

There was a consensus among the participants that there was no collaboration between the educators and the physiotherapists at the special schools. The lack of collaboration was attributed to educators not being clear on the roles and responsibilities of physiotherapist in the SIAS process and hence, they were not involved in most instances. In addition, there was lack of support structures at the level of the school, circuit and the district.

## Discussion

A study by Louw et al. (2021) highlighted distinct demographic differences among registered physiotherapists in South Africa. Their investigation revealed the majority to be white physiotherapists (55.6%), a notable female majority (82.9%), and a median age ranging from 27 years to 37 years. In contrast to the demographic characteristics in our study, the median age of participants was above the age of 40 years. Nonetheless, both studies reported a higher proportion of females (57%) (Table 1).

### Theme 1: Poor knowledge of the Screen, Identify, Assess, and Support policy

As a result of poor knowledge, educators were unable to implement the initial stage of the SIAS process of assessment. Learner Profiles were not available to screen and identify learners who may have barriers to learning. Many school educators who received their qualifications before 2014 (when the SIAS policy was launched) were not exposed to inclusive education principles and practices during their initial training (Majoko & Phasha 2018). The SIAS policy is new for them and they require support. Similarly, studies conducted among South African educators also reported similar challenges pertaining to inadequate knowledge that led to failure to complete the SIAS toolkits (Maree, Condy & Meda 2023; Pillay & Tlale 2024). It has been commonly reported that limited knowledge is attributable to policy failure (Calvano 2013; Dembe & Partridge 2011). There is growing recognition that policies rarely fail because of their design alone but rather on their implementation (Hudson, Hunter & Peckham 2019). Factors contributing to poor knowledge of SIAS policy are discussed next.

#### A lack of in-service training

Ideally, training on policy goal and implementation toolkits should precede any action tended towards policy implementation. Such training may need to be repeated to ensure that both the policy goals and implementation strategies are clearly endorsed to the relevant stakeholders (Calvano 2013; Dembe & Partridge 2011). The training for education physiotherapists occurred over 2 days and was not repeated for other staff members appointed at a later stage. Similarly, training for educators targeted only School- Based Support Teams and it was a one-time event. The cascade model of training was employed whereby the individuals who receive the formal training would be expected to train others who were not present during the original training. While this model can be cost-effective for the schools, it can also be ineffective (Jita & Mokhele 2014) as it may not adequately address the needs of all staff, leading to negative attitudes towards policy implementation (Calvano 2013). It is commonly reported that the training on the SIAS policy and its toolkits is often superficial and inadequate (Maree et al. 2023). Because of the lack of ongoing training and capacity development by the provincial Department of Education, physiotherapists took the initiative to self-acquaint themselves with the SIAS policy. This initiative demonstrates that physiotherapists applied their graduate attributes to being lifelong learners. Although physiotherapists could adapt without formal training, their understanding and application of the policy could be inconsistent. Inconsistency will not achieve the uniformity and standardisation the SIAS assessment process intends to achieve.

Addressing these training gaps and incorporating inclusive education in the initial training curriculum could be essential steps towards improving the situation (National Academies of Sciences, Engineering, and Medicine et al. 2024). Training and professional development initiatives can be introduced to enhance educators’ comprehension of physiotherapists’ roles and expertise, encouraging a collaborative approach to support learners with special needs (Jita & Mokhele 2014; Skrypnyk et al. 2020). Physiotherapists should also attempt to expand their knowledge base as part of their continuous professional development. There is a need for accredited short-learning courses to train therapists (physiotherapists, occupational therapists and speech therapists) in the education sector on the SIAS policy.

#### Fear of transfers through the Rationalisation and Redeployment policy

Physiotherapists were of the view that educators perceived implementation of the SIAS policy might activate the R&R policy, which could work against their interests. Implementing the SIAS policy would result in learners being placed according to the level of support they require, which may potentially result in a learner being transferred to either a mainstream school or a full-service school. This reassignment could lead to a decrease in school enrolment, affecting staffing levels. Educators feared that a decline in learner enrolment would make them targets for redeployment under the R&R policy and be transferred to other schools they may not prefer (Nemutandani 2004). This sentiment was also shared from a case study that explored the challenges and adaptations of educators during rationalisation and redeployment in public schools in Limpopo province, South Africa (Rapeta 2024). Educators who have been redeployed in the above-mentioned study reported that they would have preferred to resign rather than being redeployed. In another case study among South African educators, it was found that the redeployment policy caused educators to become less motivated in performing their functions optimally (Makhonza & Gobingca 2023).

Educators viewed the R&R policy in a negative light, seeing it as punitive law rather than a strategy to address human resource challenges (Mgojo 2019). This fear is likely going to lead to resistance towards implementing the SIAS policy optimally to benefit learners. Thus, effective communication is crucial. School management and authorities should clearly explain the R&R policy’s purpose and objectives, highlighting its strategic, non-punitive nature. Emphasis should be placed on how the SIAS policy aims to support the needs of the learners and improve their educational outcomes. Such communication could alleviate fears and foster a more receptive attitude among educators towards the SIAS process of assessment, ultimately benefitting learners, educators and physiotherapists.

One of the underlying issues contributing to the knowledge challenges faced by education physiotherapists and educators is the lack of inclusive education in the undergraduate teaching curriculum (Hess 2020), especially for professionals who qualified prior to the launch of the SIAS policy in the year 2014.

### Theme 2: A lack of interprofessional collaboration

Physiotherapists indicated that there was no working relationship between them and educators at schools. Physiotherapists were not involved in the assessment of learners with the SBST and referrals were delayed. These findings concur with those from Wiklund et al. (2024) in a study exploring facilitators and barriers in interprofessional collaboration pertaining to physical activity on prescription among Swedish schools. School physiotherapists in the above-mentioned study reported being isolated and often perceived as outsiders in the schools. Similarly, the study by Skrypnyk et al. (2020) investigated collaboration of teachers with specialists and parents in supporting Children with Special Needs. The specialists included school physiotherapists and reported a lack of collaboration between them and teachers. It was reported that teachers preferred to work independently. This highlights the need for improved interprofessional collaboration between physiotherapists and teachers in school settings. Contributing factors for a lack of interprofessional collaboration are discussed next.

#### A lack of role and responsibilities

The lack of involvement and understanding of the role of physiotherapists in the school contributed to their marginalisation, which was detrimental to the goal of building inclusive school environments. The expertise of various professionals, such as physiotherapists, could complement each other to meet the diverse needs of learners effectively. The lack of clear roles and responsibilities was also found to be a barrier in schools when implementing school programmes, for example, physical activity (Wiklund et al. 2024).

The absence of well-defined directives and designated responsibilities resulted in confusion and uncertainty among teams leading to an improvised approach where education physiotherapists believed they were required to undertake a wide array of tasks without precise guidance.

Hemmingsson, Gustavsson and Townsend (2007) and Paulsrud and Nilholm (2023) reported that unclear roles and responsibilities undermined the effective working collaboration in inclusive education. Teachers, therapists, and special educators often held different understandings of their responsibilities, leading to unintegrated support for learners. Commonly, factors such as limited time, power imbalances between educators and therapists, and policy misalignment further compound the challenge by restricting collaboration (Garcia-Melgar et al. 2022; Skrypnyk et al. 2020). These studies highlight the need for well-defined, shared role description and institutional support to enable meaningful collaboration.

#### A lack of support structures

The absence of the Circuit-Based Support Team meant that the SBST referred school cases directly to the district and bypassing the circuit. This gap in the referral pathway may cause delays at the district level when trying to support schools. In addition, Makhalemele (2011) and Nel et al. (2016) reported other factors that contributed towards DBST having challenges in providing support to the schools. These challenges included inadequate facilities and infrastructure to provide education support, transport to visit schools and a shortage of human resources. In another study by Motitswe (2014), it was found that time frames to support learners were long (about 4–6 months) because the DBST took a long to give feedback on cases referred to them. Consequently, the DBST is burdened with many administrative tasks, affecting its effectiveness (Mfuthwana & Dreyer 2018; Mpanza & Govender 2022). This finding underscores the need for the provincial Department of Education to release resources to strengthen the DBST to perform their duties towards supporting the SBSTs (Makhalemele & Tlale 2020).

### Limitations

While there were 35 special schools in Limpopo province, the study focused on only three that only admitted learners with physical disabilities because of the availability of education physiotherapists based at the district level and at the school level. Thus, the province itself is limited to the number of physiotherapists in the education setting. The authors used a consensus agreement approach to arrive at the conclusion of the two themes that emerged from the data. The fact that we conducted a single FGD constitutes a limitation. This is because of the low number of education physiotherapists within Limpopo province. There is the risk of social desirability bias because of group dynamics as individuals may have felt pressure to conform to dominant opinions and avoid disagreements. In addition, field observations may have influenced by the author’s viewpoint and may not have reflected participants’ behaviour.

## Conclusion

Overall, the study highlighted challenges experienced by physiotherapists in the education setting when implementing the SIAS policy to support learners with physical disabilities. Poor knowledge of the policy was the main contributing factor among educators and physiotherapists. The poor knowledge was attributable to the lack of ongoing in-service training by the employer. Thus, as a result, Learner Profiles were not completed, the working relationship between educators and physiotherapists was suboptimal because of unclear roles and definitive responsibilities towards supporting learners. It is vital to address role clarity and collaboration issues among stakeholders to enhance the implementation of SIAS policy and foster inclusive school environments. Precise directives and transparent communication about the involvement and responsibilities of physiotherapists in school committees must be established.

In addition to the limited knowledge on the policy among educators, the study identified underlying fears that negatively influences educators in performing their responsibilities. These fears stem from the Rationalisation and Redeployment policy, which would transfer educators from schools with excess staff to other schools where there is shortage as learners are moved out of special schools.

The study revealed a missing layer in the referral pathway in the SIAS policy such as the missing Circuit-Based Support Team. This missing layer in the support structure could be exacerbating issues already affecting the functioning of the DBST in supporting schools.

Thus, the findings of this study provide insights that policymakers may find helpful when considering the review and evaluation of the SIAS policy implementation.
